# A new computational method to predict transcriptional activity of a DNA sequence from diverse datasets of massively parallel reporter assays

**DOI:** 10.1093/nar/gkx396

**Published:** 2017-05-22

**Authors:** Ying Liu, Takuma Irie, Tetsushi Yada, Yutaka Suzuki

**Affiliations:** 1 Department of Computational Biology and Medical Science, Graduate School of Frontier Sciences, the University of Tokyo, Chiba, Japan; 2 Department of Bioscience and Bioinformatics, Kyushu Institute of Technology, Fukuoka, Japan

## Abstract

In recent years, the dramatic increase in the number of applications for massively parallel reporter assay (MPRA) technology has produced a large body of data for various purposes. However, a computational model that can be applied to decipher regulatory codes for diverse MPRAs does not exist yet. Here, we propose a new computational method to predict the transcriptional activity of MPRAs, as well as luciferase reporter assays, based on the TRANScription FACtor database. We employed regression trees and multivariate adaptive regression splines to obtain these predictions and considered a feature redundancy-dependent formula for conventional regression trees to enable adaptation to diverse data. The developed method was applicable to various MPRAs despite the use of different types of transfected cells, sequence lengths, construct numbers and sequence types. We demonstrate that this method can predict the transcriptional activity of promoters in HEK293 cells through predictive functions that were estimated by independent assays in eight tumor cell lines. The prediction was generally good (Pearson's *r* = 0.68) which suggested that common active transcription factor binding sites across different cell types make greater contributions to transcriptional activity and that known promoter activity could confer transcriptional activity of unknown promoters in some instances, regardless of cell type.

## INTRODUCTION

In metazoan cells, the processes of gene expression are regulated by various protein–protein, DNA–DNA interactions as well as protein–DNA interactions that involve transcription factors (TFs) binding to functional DNA segments that are pervasive in transcriptional initiation, elongation and termination. To reveal the regulatory processes of gene expression, many experimental approaches were developed by taking different biological features into account, including the ENCODE Project (1) for investigating epigenetic modifications and transcription factor binding; MPRA (2) (massively parallel reporter assays), CRE-seq (3,4) (*cis*-regulatory element analysis by sequencing) and STARR-seq (5) (self-transcribing active regulatory region sequencing) for measuring reporter activity of putative regulatory sequences; and Hi-C technology (6) for investigating the interaction of promoters and distal enhancers (7) by capturing three-dimensional chromatin structures. Additionally, c*is*-regulatory elements, which contain promoters and enhancers, regulate gene expression via TF binding, and thus, c*is*-regulatory elements have been used frequently to explore TF binding affinity in transcription processes (3,8,9).

In recent years, the dramatic increase in the number of applications of MPRA technology (2,3,8–13) produced a large body of data from *cis*-element reporter assays for different purposes, such as data for investigating genomic variants (10), distinguishing functions between promoters and enhancers (11), analyzing motifs or transcription factor binding sites (TFBSs) (8,13). MPRA is a kind of transient reporter assay in which the target sequences are cloned into reporter gene vectors and random barcodes are attached to the 3΄ end of a reporter gene to label different sequences. The target sequences to be assayed by MPRA are mainly produced in two ways: DNA synthesis (equal lengths and <200 bp) or captured chromatin segments (with different lengths) according to different purposes (2,3,8–13). The plasmid libraries are subjected to transfection, and then, the barcodes of mRNA are detected by high-throughput sequencing. In MPRA, the transcriptional activities are generally identified by the ratios of barcode counts of mRNA to the template DNA.

Several previous studies investigated *cis*-regulatory elements via conventional luciferase reporter assays (14,15). However, the throughput of luciferase reporter assays is generally up to several thousand, and therefore, computational approaches for predicting transcriptional activity were mostly used for small and high-similarity data sets. In addition, existing computational processes for analyzing MPRAs were mostly designed in customized and dedicated ways, such as summarizing SNPs (10) and testing the effects of order, orientation, and copy number for specified TFBSs or motifs (12,13). In the previous study of (2), a quantitative sequence-activity model (16) (QSAM) performed well in predicting transcriptional activities using the MPRA data sets. However, a QSAM has limited adaptability for data sets with unequal sequence lengths. Therefore, a computational method that is applicable to deciphering regulatory codes for diverse MPRA data types does not exist yet.

In various computational biology studies, diverse machine learning algorithms have been applied to construct a quantitative model to predict biological levels. These algorithms are roughly separated into ‘white-box’ algorithms and ‘black-box’ algorithms. The response functions of the white-box algorithms, which are generally constructed as a mathematical combination of predictors (or features), are obvious and understandable. In contrast, black-box algorithms hide the details of trained functions. Namely, multiple linear regression (17) (MLR), Lasso regression (18), multivariate adaptive regression splines (19) (MARS) and regression tree (20) are white-box algorithms. For these algorithms, their visible response functions would not only obtain quantitatively predicted values through data training but also allow for extraction of the computational relationship among different features of input data by analyzing the structures of response functions.

In this research, we propose a new computational method to predict the transcriptional activity of different MPRA, as well as luciferase reporter assay via combined usage of the TRANScription FACtor database (21,22) (TRANSFAC) and the computational processes of regression trees and MARS. TRANSFAC, which is a well-known eukaryotic TFBS profile database, was introduced into the proposed method to encode *cis*-element sequences into TFBS enrichment. MARS is a well-known algorithm that builds response functions through the summation of hinge functions and products of multiple hinge functions. A hinge function takes the form of *max(0,x-c)* or *max(0,c-x)*, where *c* is a constant estimated by MARS and *x* is given by explanatory variables. A regression tree is a kind of a white-box and decision tree learning algorithm that is frequently utilized both for classification and regression. The estimated result of the regression tree is demonstrated via the tree structure, which is understandable and interpretable. However, a limitation of regression tree analysis is its vulnerability to over-fit errors, and it is unreasonable to tune the tree size manually for different properties of the input data. In this study, we considered a feature redundancy-dependent formula to automatically tune the tree size for different data sets.

We demonstrate that the proposed method should be applicable to diverse MPRA (as well as luciferase reporter assay) data sets despite differences in transfected cell types (HEK293, HepG2, K562 and other tumor cell lines of human, yeast and mouse), sequence lengths (87 bp to >1300 bp), construct numbers (several hundred to >27 000) and sequence types (promoters, enhancers, designed motifs, ChIP-seq peaks and genomic variants) (Table 1).

**Table 1.** Basic information contained in the data sets

Data sets	Description	Construct lengths	Cell types	Assayed loci	#Constructs	References
Melnikov *et al*.	CRE enhancer with 10% random mutations	87 bp	HEK293	*Ex vivo*	27000	(2)
Shen *et al*.	3500 DNase I hypersensitive sites	181–703 bp (median 466 bp)	Mouse retina	*Ex vivo*	27161	(8)
Sharon *et al*.	Designed 75 yeast TFBSs	103 bp	Yeast	*In vivo*	6016	(13)
Smith *et al*.	12 liver-specific TFBSs assayed in HepG2 and mouse cells	168 bp	Mouse, HepG2	*In vivo, ex vivo*	4742	(12)
Ulirsch *et al*.	2756 SNPs assayed in GATA1 overexpression +/- K562 cells	145 bp	K562	*Ex vivo*	15733	(10)
Irie *et al*.	Promoters	755–1201 bp (median 1081 bp)	HEK293	*Ex vivo*	734	(14)
Nguyen *et al*.	253 distal enhancers and 234 promoters assayed by MPRA and STARR-seq	139 bp	Mouse cortical neurons	*Ex vivo*	3409	(11)
Landolin *et al*.	Promoters assayed in eight cell types	614–1301 bp (median 983 bp)	Ags, G402, HCT116, Hela, Hepg2, HT1080, T98G, U87mg	*Ex vivo*	4575	(15)

This paper consists of two parts. First, we developed a new computational method to predict transcriptional activity by using the method on MPRA data. Second, we deciphered the TFBSs that are active during transcriptional regulation by analyzing the response functions of MPRA data training. For that approach, the proposed method utilized the TRANSFAC database to construct biological features from DNA sequences and the computational processes for predicting transcription activities based on two white-box algorithms called regression tree and MARS. We found that our method could calculate the data fitting values as predicted values and, furthermore, the structure of corresponding response functions, which model the input features in mathematical ways. Additionally, the method could estimate the biological significance and relationship of features. Moreover, the prediction of the transcription activities by this method allowed us to estimate the transcription activities of new sequences by using known transcription activities. By analyzing the corresponding response functions, we could even obtain important information clues for the sequence design of *cis*-elements, which would further decipher the regulatory code of transcription.

## MATERIALS AND METHODS

### Data sets

We selected 10 public data sets from eight previous works (Table 1) that contain eight MPRA data sets and two luciferase reporter assay data sets. The DNA sequences and corresponding transcriptional activities were required to construct predictive functions. Among the different data sets, the sequence patterns could be roughly separated into three types: (i) target sequences that were designed by introducing random mutations into an original sequence (2); (ii) artificial sequences in which different permutations of motifs or TFBSs of interest were inserted into template sequences (12,13) and (iii) chromosomal segments selected based on prepared criteria (8,10,11). The transcriptional activity of diverse sequence patterns was measured in different cell lines and different species, including HEK293, HepG2, K562 and other human tumor, yeast and mouse cell lines. Different experimental designs led to having target sequences with both equal and unequal lengths within the same data set, and the sequence lengths across 10 data sets ranged from 87 bp to >1300 bp. The library sizes also had a wide range, from several hundred constructs to several tens of thousands. In contrast to MPRA, data sets of luciferase reporter assays generally have relatively longer sequences and smaller library sizes (Table 1).

The data set of Melnikov *et al*. (2) has the transcriptional activities of 27 000 mutant CRE (cAMP response element) enhancers in HEK293 with the equal length of 87 bp. The study of Shen *et al*. (8) employed MPRA for 3500 DNase I hypersensitive sites (DHS) in mouse retina with unequal sequence lengths (181–703 bp). Designed sequences were used by Sharon *et al*. (13) to investigate the contributions of different TFBS properties, such as location, number and orientation, to the transcriptional activities of 75 yeast TFBSs via MPRA. The studies of Irie *et al*. (14) and Landolin *et al*. (15) both assayed promoter sequences using luciferase reporter assays, and their sequence lengths ranged from several hundred bp to >1 kb. Several studies applied MPRA under different experimental conditions: the study by Smith *et al*. (12) assayed the designed sequences of 12 liver-specific TFBSs inserted into template sequences in mouse and HepG2 cells; a study by Ulirsch *et al*. (10) employed MPRA for 2756 genomic variants of red blood cells (RBC) in K562 cells and GATA1 overexpression (OE) K562 cells. For the data sets with double experimental conditions, we used the proposed method to estimate the transcriptional activities under different experimental conditions to detect experimental condition-specific features. In addition, there are two data sets that contain reporter assays under multiple (>2) experimental conditions: the data set of Nguyen *et al.* (11) has the transcriptional activities in mouse cortical neurons and KCL (potassium chloride)-stimulated mouse cortical neurons, obtained using both MPRAs and STARR-seq; the data set of Landolin *et al*. has the transcriptional activities of 4575 promoters in 8 tumor cell lines (Ags, G402, HCT116, Hela, Hepg2, HT1080, T98G, U87mg), obtained using luciferase reporter assays. For the data sets with multiple (>2) experimental conditions, we constructed integrated predictive functions across different conditions.

### Data pre-processing

**Figure 1.**
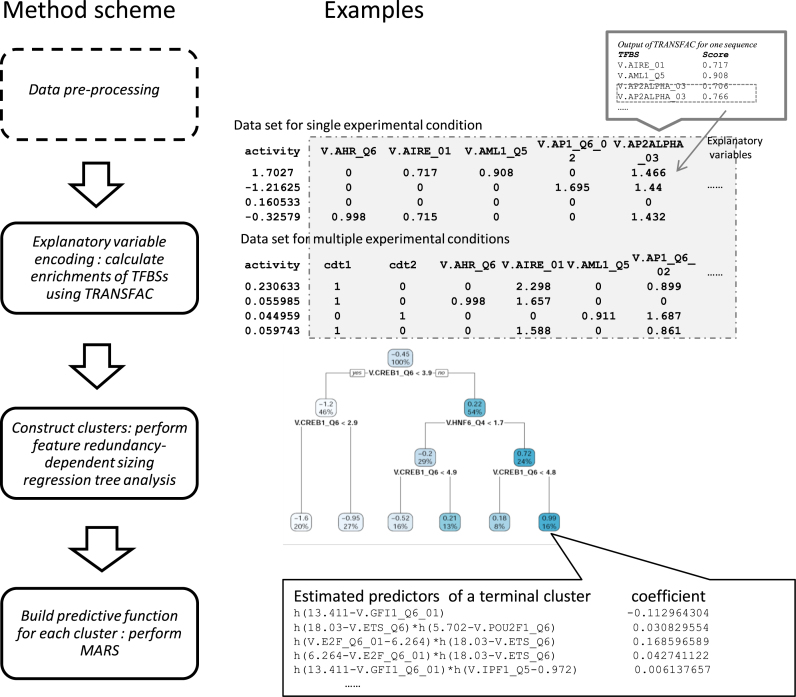
Schematic of the proposed method and examples. (Left) The method consists of four steps: (1) the data were pre-processed; (2) the sequences were characterized as TFBS enrichment scores through the use of TRANSFAC database and used as the explanatory variables, while the explanatory variables of different sequences were assembled into an explanatory variable matrix; (3) the explanatory variable matrix and the corresponding transcriptional activity were input into a feature redundancy-dependent sizing regression tree, which has a proposed feature redundancy-dependent formula to enable adaption to diverse data sets to construct clusters; and (4) MARS was used to construct predictive functions for individual clusters estimated in the third step. Detailed procedures for each step are described in Material and Methods. (right) The explanatory variables were determined by the summation of the PWM scores of each kind of TFBS. For data with multiple experimental conditions, different conditions were encoded into binary codes 0–1 and were added to the matrix of TFBS enrichment scores. Regression tree analysis separated the whole data set into different clusters according to the logic conditions shown in the nodes of the tree. Predictors and corresponding coefficients of predictive functions were estimated by applying MARS to each cluster.

The sequences and corresponding transcriptional activity within data sets were required for training predictive functions. The forms of transcriptional activity are diverse across different data sets because they are produced by different studies of MPRAs, and we estimated the transcriptional activity using the same method. The transcriptional activity of MPRAs was calculated by the log_2_ ratios of the mRNA tag counts to DNA tag counts of identical barcodes (except for the data set of Sharon *et al*.; see Supplemental materials), and the transcriptional activities were identified by the log_2_ expression of the reporter gene using luciferase reporter assays. Moreover, the experimental precision of individual data sets was estimated by experimental replicates, and the relatively inaccurate and irregular samples were removed from the data sets (Figure 1 and Supplemental materials).

### Explanatory variable encoding

The sequence patterns of different data sets showed a great deal of variety (Supplemental Figure S1) because they were designed for diverse purposes, and we sought to encode sequence patterns in a uniform and compact way such that the variable complexity is not altered by sequence lengths and sequence similarities. Here, we utilized a database to characterize sequence patterns.

TRANSFAC (21,22) (TRANScription FACtor database), which is a well-known eukaryotic TFBS profile database, was introduced into this study to encode DNA sequences into TFBS enrichment scores as explanatory variables (Figure 1). For each sequence, TFBS enrichment scores were calculated by the summation of the Position Weight Matrix (PWM) scores of the corresponding TFBS, and the TFBS enrichment scores of all the sequences were arrayed as an explanatory variable matrix.

\begin{eqnarray*} &&\ {\rm{TFBS}}\,{\rm{enrichment}}\,{\rm{scor}}{{\rm{e}}_{ij}} \nonumber \\ &&= \mathop \sum \nolimits_{\rm{k}} {\rm{PWM}}\,{\rm{matrix}}\,{\rm{scores}}\,{\rm{of}}\,k - {\rm{th}}\,{\rm{TFBS\ }}i{\rm{\ in}}\,{\rm{sequence\ }}j\end{eqnarray*}

The TRANSFAC matrices of fungi were utilized for the data set of Sharon *et al*.; matrices of a liver-specific profile were used for the data set of Smith *et al*.; and non-redundant vertebrate matrices were used for the other data sets. All the matrix profiles with cut-offs were set to minimize the false negative rates.

A high false positive rate is a common limitation of computational approaches based on the TRANSFAC database, and we wanted to develop a simple and interpretable predictive method with a small number of predictors that make significant contributions to transcriptional activity. Therefore, only the sequence feature of TFBS enrichment scores was adopted, and we ignored other relatively trivial features, such as TFBS position and orientation (Figure 1).

Several reporter assays were applied to the same sequence library under more than two experimental conditions, such as assaying in different cell lines (15) and different types of transcription-associated activity (11). For data sets that contain multiple experimental conditions, we considered additional explanatory variables to characterize different experimental conditions. The additional variables were encoded into binary code (0–1) to represent different conditions (e.g. cell types) and for combination with the corresponding TFBS enrichment scores as an explanatory variable matrix (Figure 1).

### Feature redundancy-dependent sizing regression tree

After constructing the explanatory variable matrix, regression tree analysis was performed to assemble samples into different clusters. The building process of a regression tree consists of two steps: (i) the data are recursively separated into two clusters (binary tree) until the clusters either reach a pre-defined size (in the R package ‘rpart,’ the parameter is named *minbucket*) or until no improvement can be made; (ii) cross-validation is used to trim back the tree. For different data sets that do not have similar feature patterns (Supplemental Figure S1), it is not sufficient to control the tree size only by splitting the tree such that the cross-validation increases in the second step, as in the conventional regression tree.

In this study, a feature redundancy-dependent formula was proposed to specify the minimum number of samples in any terminal cluster of the regression tree. In the R package ‘rpart,’ we used as the formula to specify the *minbucket* parameter (see also Supplemental materials).

The parameter of *minbucket* was determined by:

\begin{eqnarray*} &&minbucket\nonumber \\ && = {\rm{\ variation\ parameter*number\ of\ observations}}\end{eqnarray*}

\begin{eqnarray*} &&variation\ parameter \nonumber\\ &&= \frac{{{2^{ - {\rm{Proportion\ of\ variance\ of\ the\ first\ principal\ component}}}}{\rm{*}}1{\rm{e}} + 07}}{{{\rm{number\ of\ observation}}{{\rm{s}}^2}}}\ \end{eqnarray*}

The proportion of variance of the first principal component could be calculated by principal component analysis (23) (PCA). The first principal component (PC1) is defined by the first eigenvector of the covariance matrix of features, and the variance of PC1 indicates how redundant all the features are. Here, we estimated the redundancy of TFBS enrichment scores (Table 2), and we found that the data set of Melnikov *et al*. had the smallest value of proportion of variance of PC1. This probably occurred because the data set has different sequences with non-biased random mutations and exhibited the lowest feature redundancy. This value was transformed into a negative exponent form and divided by the squared number of observations, which indicates that the relatively larger data sets should build more complex trees because a large number of samples is easy to assemble into compactly terminal clusters. In other words, data sets with a small number of samples (e.g. the data set of Irie *et al*.) do not split the data set into different clusters because the features are sparse and vulnerable to over-fitting (see Table 2; the *minbucket* of Irie *et al*. is larger than its number of observations, hence no clustering). Other constants in the formula of the *variation parameter* are used to make the *variation parameter* load within a desired scale.

**Table 2.** Values of ‘*Variation parameter*’ and ‘*minbucket*’ for different data sets

Data sets	Proportion of PC1	*Variation parameter*	*minbucket*
Nguyen *et al*.	0.37	0.04	566.06
Melnikov *et al*.	0.10	0.01	354.31
Shen *et al*.	0.47	0.01	266.57
Landolin *et al*.	0.78	0.00	159.29
Iriel *et al*.	0.71	11.33	8318.43
Ulirsch *et al*.	0.48	0.03	456.04
Smith *et al*.	0.35	0.35	1657.98
Sharon *et al*.	0.50	0.20	1177.21

### MARS performed in each cluster

The regression tree analysis separated whole sample libraries into different clusters, and then, explanatory variables of subpopulations and the corresponding transcriptional activity were input into MARS for training predictive functions of individual clusters (Figure 1). In this study, we constructed predictive functions of transcriptional activity in each terminal cluster using MARS as follows:

\begin{equation*} \begin{array}{l} {\rm transcriptional\ activity}_{\rm j}\\ = \sum {\rm c}^{\prime}*{\rm h(TFBS\ enrichment\ score}_{i^{\prime}j})*{\rm h(TFBS\ enrichment\ score}_{i^{\prime\prime}j})\\ + \sum {\rm c}^{\prime\prime}*{\rm h(TFBS\ enrichment\ score}_{i^{\prime\prime\prime}j}) + {\rm c} \end{array}\end{equation*}

Here, ${\rm{TFBS}}\;{\rm{enrichment}}\;{\rm{scor}}{{\rm{e}}_{i^{\prime}j}}$ represents the *i*th TFBS enrichment score in DNA sequence *j*, and h(–) is a hinge function and *c* is a coefficient, both estimated by MARS. In the training process of MARS, MARS recursively adds a new predictor that reduces the sum-of-squares residual (RSS) error and removes the least effective predictor by generalized cross validation (GCV) that penalizes the number of predictors to avoid over-fitting (19). The predictors, for which TFBS enrichment scores were characterized by hinge functions, captured the switch-like features that TFBS enrichments along a sequence larger (or smaller) than a scale (estimated by MARS) make contributions to transcriptional activity. Moreover, the products of the hinge functions of two TFBS enrichment scores indicate the estimated interactions between corresponding TFBSs.

## RESULTS

### General descriptions

In this study, we propose a new computational method to predict transcriptional activity using DNA sequences and the corresponding transcriptional activity using MPRAs. The method consists of four steps (Figure 1): (1) the data were pre-processed; (2) the TRANSFAC database was introduced and the sequences were characterized as TFBS enrichment scores and used as the explanatory variables, while the explanatory variables of different sequences were assembled into an explanatory variable matrix; (3) the explanatory variable matrix and the corresponding transcriptional activity were input into a feature redundancy-dependent sizing regression tree, which has a proposed feature redundancy-dependent formula to enable adaption to diverse data sets to construct clusters and (4) MARS was used to construct predictive functions for individual clusters estimated in the third step. The detailed procedures for each step are described in Materials and Methods.

**Figure 2.**
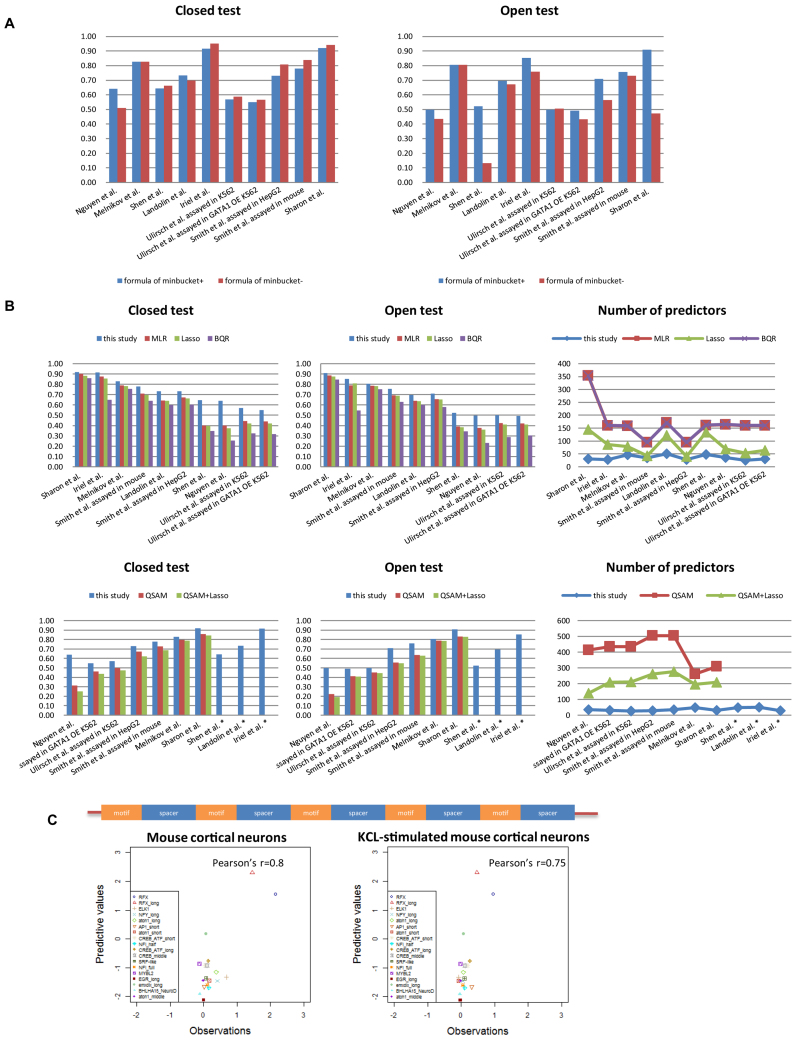
The predictive precision and the corresponding number of predictors of the proposed method and compared methods. The bar graphs show the correlation coefficients between predictive values and experimental values for different data sets of the closed test and open test (100-fold cross-validation). The number of predictors of the proposed method indicates the maximum number of predictors among all terminal clusters estimated by regression trees (Figure 1). (**A**) The correlation coefficients between the predictive values and experimental values of the proposed method with and without using the ‘*minbucket*’ formula. (**B**) The correlation coefficients between the predictive values and experimental values of other machine learning algorithms (MLR, Lasso regression and BQR) and QSAMs. The data sets with *indicate that QSAMs could not be applied to the corresponding data set. (**C**) (Upper) Designed sequence patterns of the 18 motifs selected by (11). The corresponding motif repeats were separated by three types of 11-bp spacers. (Lower) Scatter plots between the predictive values and observations of individual motifs for assays under different experimental conditions in mouse cortical neurons and KCL-stimulated mouse cortical neurons. The obtained correlation coefficients between the predictive values and experimental values of the 18 individual motifs were approximately 0.75 and 0.80 for KCL-stimulated mouse cortical neurons and mouse cortical neurons, respectively.

To demonstrate the usability, we applied the proposed method to 10 different data sets and obtained predictive functions that consisted of 16–50 predictors for each cluster and averaged 33.1 predictors across all data sets. The correlation coefficients between predictive values and experimental values for individual data sets were ∼0.5–0.9 (Figure 2 and Supplemental table 1), and the correlation coefficients of the open test, which were evaluated by 100-fold cross-validation, approached the closed test. Based on the series of evaluations, we concluded that the proposed method should provide useful means to investigate the primary DNA sequence encoding the potential transcriptional activities under various conditions.

### Construction and evaluation of the method

#### Construction of the method

The improvements of predictive precision by introducing the feature redundancy-dependent formula of *minbucket* into a conventional regression tree are shown in the results (Figure 2A). The proposed formula of *minbucket*, which aims to balance the over-fit and under-fit of predictive function training, was added to conventional processes so that regression tree analysis could be adapted to different data types. For the data sets of Shen *et al*. and Sharon *et al.*, the conventional regression tree analysis fell into over-fitting, and the usage of the *minbucket* formula increased the performance of 100-fold cross-validation approximately 3.0 and 0.9 times, respectively. For the data sets of Nguyen *et al.*, Irie *et al.*, Ulirsch *et al*. assayed in GATA1 OE K562 cells and Smith *et al*. assayed in HepG2 cells, the formula of *minbucket* also contributed to increasing the performance of the open tests by ∼10–26% (Figure 2A).

The proposed method was constructed for estimating the quantitative scale of transcriptional activity, and we attempted to quantitatively validate the predictive results, not only by the 100-fold cross-validation method but also by predicting transcriptional activity for new data from independent experiments.

Other MPRAs were applied by (11) to measure the transcriptional activity of 18 selective motifs (6–17 bp) under the same experimental conditions as the data set in Nguyen *et al.*, and the artificial sequences were designed so that corresponding motif repeats were separated by three types of 11 bp spacers (Figure 2C). We calculated the average activity of the motif sequences with different spacers as the motif activity to reduce the influence of the spacers (see also Supplemental materials and Supplemental Figure S5). To evaluate the predictive precision of the proposed method for new data, we used the predictive functions for Nguyen *et al*. data to predict the transcriptional activity of the 18 motifs. Because the predictive functions for Nguyen *et al*. data integrated multiple experimental conditions, we could predict the activity both in common mouse cortical neurons and in KCL-stimulated mouse cortical neurons using MPRAs. We predicted 18 individual motifs and obtained correlation coefficients between predictive values and experimental values of approximately 0.75 and 0.80 (Figure 2C) for the assays in KCL-stimulated and normal cells, respectively.

However, we also found that the correlation coefficients between observations and predictive values were influenced by the high transcriptional activities of two RFX (regulatory factor X) motifs (‘RFX’ and ‘RFX_long’ in Figure 2C), and the correlation coefficients decreased to approximately 0.2 and 0.02 for the assays in KCL-stimulated and normal cells if the two RFX motifs were removed (see also Supplemental Figure S7). This probably occurred because there are different feature patterns between the trained data set (genomic segments) and predicted data set (designed sequences), and the relatively low sensitivity of the proposed method also limits the performance for samples that have low activities. For example, in contrast, the predictive precision of the five motifs (‘ELK1’, ‘NFY_long’,‘atoh1_long’,‘atoh1_short’ and ‘NFI_half’ in Figure 2C and Supplemental Figure S7) that have the highest transcriptional activities in mouse cortical neurons if the two RFX motifs were removed was ∼0.56 (Pearson's *r*). This result suggests that, albeit with relatively low sensitivity, the proposed method could quantitatively predict the transcriptional activity of new sequences using predictive functions that were estimated based on known data sets.

#### Comparisons with machine learning algorithms

Conventional computational approaches that focus on decoding regulatory codes are mostly considered at either the motif resolution or single nucleotide resolution level. An analysis of the motif resolution commonly uses motif similarity scores as explanatory variables, and for an analysis at single-nucleotide resolution, individual nucleotides at different positions are encoded into explanatory variables. Regarding method comparisons, the two aspects of motif resolution and single-nucleotide resolution were taken into account.

Here, we constructed three predictive methods at motif resolution using different machine learning algorithms that are widely applied in bioinformatics. MLR (17) and Lasso regression (18) are both linear models that could be used to investigate the linear relationship among different explanatory variables. Lasso regression could also be used for feature selection. Bayesian quantile regression (24) (BQR) is a kind of quantile regression that estimates the quantiles of response variables rather than the means, as MLR does. The explanatory and response variables that were input into different algorithms were the same as for the proposed method (Materials and Methods).

For all the data sets, the proposed method exhibited better performance than MLR, Lasso regression or BQR (Figure 2B and Supplemental Table S1). The correlation coefficients between the experimental values and predictive values of the proposed method were superior to those for MLR, Lasso and BQR at a maximum of 60% (both data sets of Shen *et al*. and Nguyen *et al*.), 72% (data set of Nguyen *et al*.) and 151% (data set of Nguyen *et al*.), respectively. The average predictive precision of this study across different data sets was improved by 22%, 26% and 51% in contrast to MLR, Lasso and BQR, respectively. Additionally, the maximum number of predictors of terminal tree clusters for individual data sets was much smaller (average of 2.3–4.7 times smaller) than those for MLR, Lasso and BQR.

Regarding the open test of 100-fold cross-validation, the average correlation coefficients increased by 14%, 16% and 43% for all data sets compared to MLR, Lasso and BQR, respectively.

#### Comparisons with QSAMs

A QSAM (16) is a computational model for sequence pattern recognition at single-nucleotide resolution, and in the former study of (2), they considered QSAMs to analyze functional elements for their MPRA data. Here, we constructed two QSAMs for the MPRA data sets that have equal sequence lengths (Figure 2B and Supplemental Table S1).

We could see that the proposed method also has better predictive precision with a much smaller number of predictors than the QSAM or QSAM combined with the methods of Lasso (see also Supplemental materials). Compared to the two QSAMs, the proposed method improved the average predictive precision by 24% and 35%, respectively.

For the open test, we obtained an increased average predictive precision of 30% and 37% relative to the QSAM and QSAM combined with Lasso, respectively. QSAMs encoded sequence patterns at the single nucleotide level, and thus, we obtained 3 times the number of variables. In this study, the QSAMs required, on average, 12.3 times the number of predictors as the new proposed method, and after variable selection using the methods of Lasso with restriction of model fitting, there were, on average, 6.5 times the number of predictors compared to the proposed method.

#### Re-evaluations of the proposed method compared to previous studies

A previous study (2) included a computational model based on a linear QSAM to predict the transcriptional activity of the Melnikov *et al*. data set and obtained a correlation coefficient of ∼0.79 for the closed test between predictive values and experimental values with 261 predictors. Another previous study (14) proposed a computational method for predicting the transcriptional activity using luciferase reporter assays and obtained correlation coefficients of the closed test and open test of 0.85 and 0.83, respectively, for the data set of Irie *et al*. They also applied their model for individual cell types to the data set of Landolin *et al*. and obtained an average correlation coefficient of approximately 0.6 with approximately 167 predictors. In this study, we obtained better performance with a much smaller number of predictors compared to the predictors reported in these previous studies (Figure 2 and Supplemental Table S1).

### Application of the method to investigating candidate-active TFBSs

As described above, we constructed and evaluated the performance of the proposed method on global and computational scales. Then, we attempted to examine whether the mathematical correlation observed above could capture the biological relevance. For this purpose, we analyzed the individual results estimated by the proposed method for the biological details of different data sets. We conducted the evaluations using the following three approaches, in which we attempted comparison of the methods and re-interpretation of the previous studies.

#### Characterizing candidate-active TFBSs via tree structures

**Figure 3.**
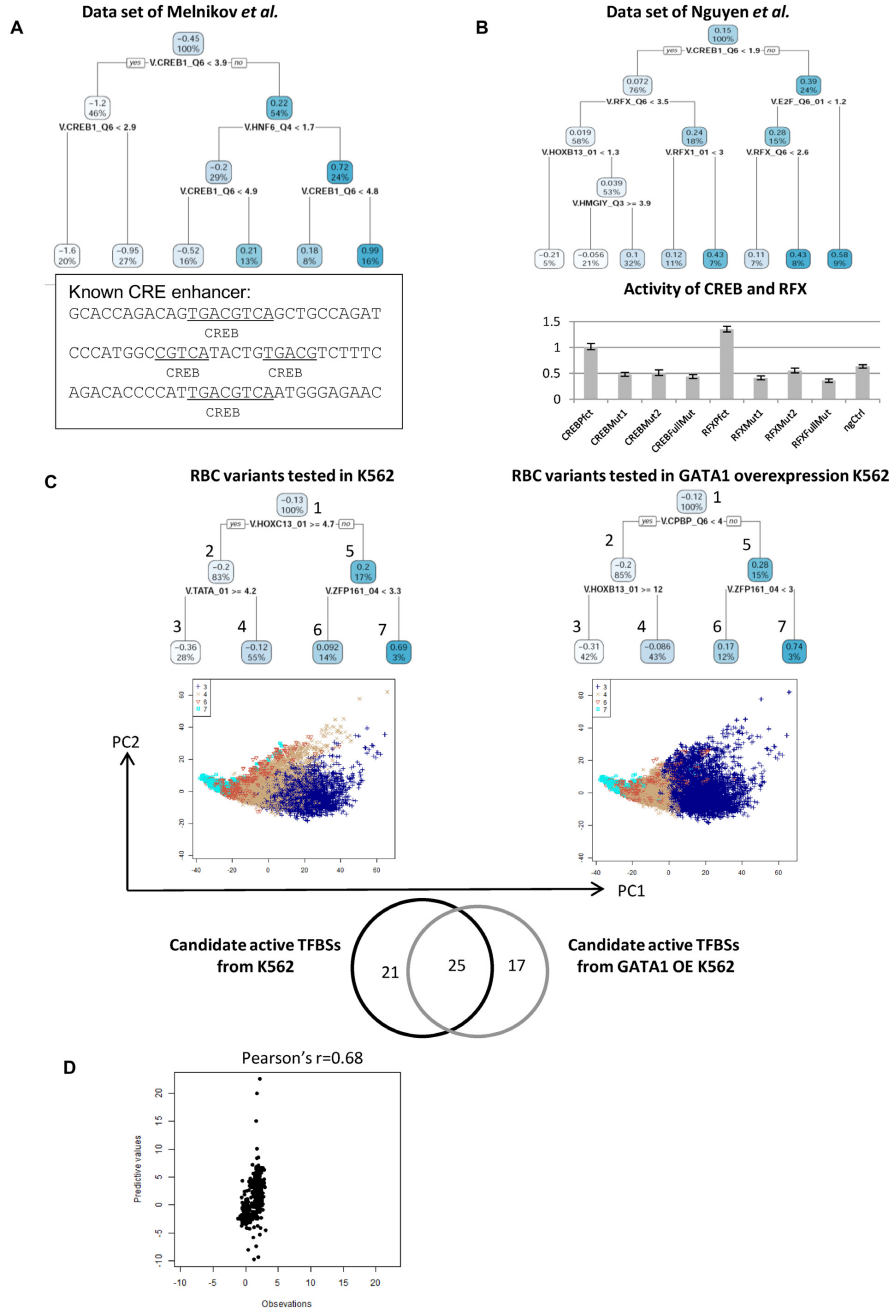
Applications of the proposed method for investigating candidate-active TFBSs. The values shown in each cluster of the regression tree indicate the average activity among samples within the corresponding cluster, and the percentages represent the sample proportions in the cluster. (**A**) (Upper) Candidate-active TFBS tree of the data set of Melnikov *et al*. (Lower) Known TFBSs in CRE enhancer described in (2). (**B**). (Upper) Candidate-active TFBS tree of the data set of Nguyen *et al*. (Lower) The average transcriptional activity of perfect motifs, two types of 2-bp mutant motifs, full mutant motifs of CREB and RFX and negative controls described in the study by Nguyen *et al*. ((11); Supplemental Materials). (**C**) (Upper) Candidate-active TFBS tree of the data set of Ulirsch *et al*. assayed in K562 cells and Ulirsch *et al*. assayed in GATA1 OE K562 cells. (Middle) Projection of TFBS enrichment scores onto PC1 and PC2 calculated by PCA for the data sets of Ulirsch *et al*. assayed in K562 cells and assayed in GATA1 OE K562 cells. The cluster numbers in the PCAs were identical to the index numbers of clusters shown in the candidate-active TFBS trees. (Lower) Frequencies of TFBSs that were selected by the predictive functions of Ulirsch *et al*. assayed in K562 and GATA1 OE K562 cells. (**D**) Plots between predictive values for the data set of Irie *et al*. estimated by the predictive functions for the Landolin *et al*. data set and observations from the Irie *et al.* data set.

The MPRAs of Melnikov *et al*. measured the transcriptional activity of mutant CRE enhancers, which were designed by introducing 10% random mutations into wild type 87 nt CRE enhancers. From the estimated results for the data set of Melnikov *et al.*, we found that the TFBS of the CREB (cAMP response element binding protein) occurred under almost all tree splitting conditions (4/5), and the transcriptional activity increased along with CREB enrichment score (Figure 3A). Known active TFBSs along the CRE enhancer were four non-overlapping CREB sites, as described in (2), and we obtained a similar result. Furthermore, the root split condition of the TFBS tree represents whether CREB enrichment scores are larger than 3.9. In this study, enrichment scores of exactly 3.9 indicate that the copy number of CREB is 4 (the cut-off of V.CREB1_Q6 to minimize false negative rates is 0.866). The regression tree just captured the consensus copy number of CREB, and it also suggested that *de novo* CREBs, which were generated by random mutations (Figure 3A), also make large contributions to transcriptional activity (Figure 3A; the average transcriptional activity increased from –1.2 to 0.22 with the occurrence of *de novo* CREB, two sided *t*-test *P*-value < 2.2e–16).

The study of Nguyen *et al*. (11) selected genomic segments bound by the coactivator of CREBBP (CREB binding protein), and reporter assays were conducted under four experimental conditions, including both promoter assays (MPRA) and enhancer assays (STARR-seq) applied in normal mouse cortical neurons and in KCL-stimulated mouse cortical neurons. The analysis results reported by (11) found that the TFBSs of CREB and RFX produced both strong promoter activity and enhancer activity (Figure 3B and Supplemental materials). Different from CREB and RFX, they also found that the TFBS of AP1 (activator protein 1) bound preferentially for enhancer activity. The predictive functions of Nguyen *et al*. estimated through the proposed method were constructed for multiple experimental conditions, and in the candidate-active TFBS tree, CREB occupied the root, which suggests that CREB makes the highest contribution to transcriptional activity across different assay types and stimulated conditions (Figure 3B). The TFBS of RFX occupied three splits among all seven regression trees, which implies that RFX has strong activity across different conditions. Furthermore, from the predictive functions of Nguyen *et al.*, we found that AP1 is one of eight candidate TFBSs that showed enhancer activity preferences (Supplemental Table S2). According to the above results, the predictive functions for Nguyen *et al*. were estimated by the proposed method, and the results are consistent with other independent studies.

#### Distinguishing candidate-active TFBSs under different experimental conditions

In the previous study conducted by Ulirsch *et al*. (10), MPRAs were applied in normal K562 and in GATA1-overexpressing K562 cells for 15 733 sequences selected from 2756 genomic variants of RBC. We built predictive functions for the data sets of Ulirsch *et al*. obtained from assays in both normal and GATA1 OE K562 cells. In the two TFBS trees estimated for different experimental conditions (Figure 3C), all the tree split determinations were different, and only one TFBS (V.ZFP161_04) appeared in both trees. These findings suggest that the mainly transcriptional processes of the target sequences significantly changed because of GATA1 overexpression.

The associations between GATA1 and TFBSs (CPBP/KLF6, HOXB13 and ZFP161) that appeared in the regression tree of Ulirsch *et al*. assayed in GATA1 OE K562 cells were unknown, and thus, we considered the candidate-active TFBSs selected by the estimated predictors. In the predictive functions of all tree terminal clusters, 46 candidate TFBSs were selected for normal K562. Regarding the predictors for the data set of Ulirsch *et al*. assayed in GATA1 OE K562 cells, 42 TFBSs were selected, and 17 of these did not overlap with the selected TFBSs of Ulirsch *et al*. assayed in K562 cells. These findings suggest that the 17 TFBSs probably led to GATA1 overexpression-responsive activity, and 10 TFBSs of the 17 were reported to associate or directly interact with GATA1 (Table 3).

**Table 3.** (Left) Candidate TFBSs interacting with GATA family transcription factors that were estimated by the predictive functions (see also Supplemental Table S4) for Ulirsch *et al*. assayed in GATA1 OE K562 cells only. (Right) Seventeen selected TFBSs from the predictive functions of Ulirsch *et al.* assayed in GATA1 OE K562 cells that did not overlap with the selected TFBSs of Ulirsch *et al.* assayed in K562 cells (see also Figure 3C)

TFBSs of interaction with V.GATA_Q6 estimated by proposed model	Description or main binding proteins	Previous reports about biological associations or interactions with GATA family	GATA1 overexpression responsive TFBSs estimated by proposed model	Description or main binding proteins	Previous reports about biological associations or interactions with GATA1
V.AP1_Q6_02	AP1	(28)	V.AP1_Q6_02	AP1	(28)
V.COE1_Q6	COE1(EBF1)	(35)	V.BBX_04	Bbx	-
V.CREBP1_01	CREB-binding protein	(36)	V.COE1_Q6	COE1(EBF1)	-
V.HOXC13_01	HOXC13	-	V.CREB1_Q6	CREB1	(36)
V.RBPJK_01	RBPJ(RBPJK)	(30)	V.CREBP1_01	CREB-binding protein	(36)
V.REST_Q5	REST	-	V.CTCF_01	CCCTC-binding factor	(27)
V.RREB1_01	RREB-1	(29)	V.EBOX_Q6_01	E-box (enhancer box)	(32)
V.TATA_01	TATA-binding protein (TBP)	(34)	V.GRE_C	GR (glucocorticoid response element)	(26)
			V.HDX_01	Hdx	-
			V.HIF1A_Q6	HIF1A	(25)
			V.HOXD12_01	HOXD12	-
			V.IRX2_01	Irx2	-
			V.MUSCLEINI_B	Muscle initiator	-
			V.MYB_05	c-myb	(33)
			V.NKX25_Q6	Nkx2-5	(31)
			V.POU2F1_Q6	POU2F1	-
			V.RREB1_01	RREB-1	(29)

Moreover, there are eight candidate TFBSs interacting with GATA family transcription factors that were estimated by the proposed method for Ulirsch *et al*. assayed in GATA1 OE K562 cells, and half of these TFBSs (4/8) intersected with the 17 candidate GATA1 overexpression-responsive TFBSs. Additionally, 6/8 TFBSs interacting with the GATA family were reported by several previous studies (25–36) (Table 3 and Supplemental Table S3).

For another data set, in the study of Smith *et al*. (12), the transcription activities of 4742 sequences with 12 liver-specific TFBSs, in which the sequences were inserted into template sequences according to pre-defined rules, such as copy numbers and permutations, were assayed in HepG2 and mouse cells. The regression trees of the Smith *et al*. data set only have one root, which means that no clustering was performed according the proposed feature redundancy-dependent formula for conventional regression trees. We analyzed the TFBS frequencies selected by the response functions of MPRA in HepG2 and mouse cells and found that the motifs bound by the TFs of FOXA1, FOXA2, HNF-1A, HNF-4A and HNF-1B showed a difference between humans and mice (Supplemental Table S5). For example, according to the estimated response function, there are four TFBSs that are mainly bound by HNF-1A. The TFBS of ‘V.HNF1_C’ was bound by HNF-1A in human HepG2 cells. In contrast, the TFBSs of ‘V.HNF1_01’ and ‘V.HNF1_Q6_01’ were bound in mice; and the TFBS of ‘V.HNF1A_01’ was bound in both species by HNF-1A. Three of the five TFs (FOXA2, HNF-1A and HNF-4A) have diverged binding events between humans and mice that were reported by a previous study (37). These results suggest the proposed method could distinguish candidate-active TFBSs under different experimental conditions, such as species-specific TFBSs.

#### Common active TFBSs across different cell types make more contributions to transcriptional activity

The study of Landolin *et al*. (15) measured 4575 promoters across 8 tumor cell lines (Ags, G402, HCT116, Hela, Hepg2, HT1080, T98G, U87mg) using a luciferase reporter assay, and another similarly independent study of Irie *et al*. measured the transcriptional activity of promoter sequences in HEK293 cells. In this study, we considered that if most TFBSs perform similar activities across different cell types, then it might be possible to predict the transcriptional activity of unknown promoters based on the transcriptional activity of known promoters despite the assays being performed in different cell types.

We then attempted to predict the transcriptional activity of Irie *et al*. sequences using the predictive functions estimated for the data set of Landolin *et al*. by setting the explanatory variables encoded by cell type at 0. These two data sets were reported by different previous studies, and the selected promoters are also different. The correlation coefficient between the predictive values and experimental values of the Irie *et al*. data set is ∼0.68 (Figure 3D), which is close to the correlation coefficients of 100-fold cross-validation within the data set of Landolin *et al*. (that is, 0.7; see Supplemental Table S1). Regarding the candidate TFBSs selected by the predictive functions of the two data sets, 8/18 selected TFBSs of the Irie *et al*. data set are exactly consistent with the selected TFBSs that occur > = 5 times in the predictors within the predictive functions estimated for the Landolin *et al*. data set (Supplemental Figure S6).

To investigate the candidate-active TFBSs that performed different regulatory mechanisms in HEK293 and 8 tumor cell lines, we picked the sequences of the 5% most over-estimated and the 5% most under-estimated by the predictive functions for the Landolin *et al*. data set, and we found 13 TFBSs in which the fold changes of their enrichments were > = 2 compared to the 10% best fitted sequences (Table 4). There are several TFs, such as EGF1, HIF1A, the E2F family and NANOG, that play different regulatory roles in tumor cells (38–41), and it is also known that SP1 expression levels are higher in cancer cell lines than in normal cells (42). These findings suggest that cell line-specific TFBSs definitely make contributions to transcriptional activity, especially for samples that were not estimated well using predictive functions based on other cell lines. However, common active TFBSs across different cell types make higher contributions to transcriptional activity, and known promoter activity could be used to predict unknown promoters to some ex tent, regardless of cell type.

**Table 4.** TFBSs in which the fold-change of enrichments were > = 2 between the 10% best predicted samples and the 10% worst predicted samples by the predictive functions of Landolin *et al*.

TFBS label	Main binding proteins
V.AHR_Q6	AhR
V.E2F_Q6_01	E2F family
V.EGR1_Q6	EGR-1
V.HIF1A_Q6	HIF-1alpha
V.MAZ_Q6_01	MAZ
V.MAZR_01	MAZ related factor
V.MECP2_02	MECP2
V.NANOG_01	Nanog
V.RNF96_01	RNF96 (TRIM28, KAP1)
V.SP1_Q6_01	Sp1 family
V.SP100_04	Sp100
V.ZFP161_04	ZF5
V.ZNF333_01	ZNF333

## DISCUSSION

In this research, we proposed a new computational method based on regression tree analysis and MARS for predicting transcriptional activity via corresponding sequences. The proposed method is applicable to diverse MPRAs, as well as luciferase reporter assays, despite the different cell lines, different sequence lengths, different numbers of constructs and different sequence origins of the experimental data (Table 1). To enable adaptation to diverse sequence patterns, we considered a feature redundancy-dependent formula to control the sizes of regression trees for individual data sets. In the proposed method, the TRANSFAC database was introduced, and sequences were characterized according to TFBS enrichment scores. However, the high false positive rate is a limitation of searching for TFBSs using motif-finding tools. Thus, we attempted to reduce the false positive rate and obtained a much smaller number of predictors in the final predictive functions than were obtained through other methods. This computational method could be applied not only to analyze candidate-active TFBSs in transcriptional processes based on given reporter assays but also to provide information for the sequence design of corresponding promoters or enhancers.

Generally, conventional methods of QSAMs are only applicable for data sets with equal sequence lengths and have a preference for high-similarity sequences because it is difficult to interpret the QSAMs unless they are derived from data sets of mutant samples of identical sequence. Compared to QSAMs, the proposed method performed better with a smaller number of predictors, and it could be applied to diverse data sets. However, the proposed method does not have high enough sensitivity that it could model single-nucleotide substitutions the way that QSAMs can.

According to the predictive precision of different data sets, we found that the proposed method performed as well as other computational models altered with TF binding-dependent transcriptional complexity. For example, the data set of Sharon *et al*. is the data set with the best predictions for all of the mentioned approaches, and the sequences of this data set were simply designed by target TFBSs being inserted into template sequences according to several defined rules, such as positions, copy numbers and orientations. Therefore, the data sets that were predicted well usually have simple sequence patterns.

The proposed method, as well as other methods, resulted in limited performances for intrinsically complex transcriptional processes, such as the interferon beta (IFN-beta) enhancer (the correlation coefficient is approximately 0.23; MPRA data from (2)), which constructs the transcriptional complex into an enhanceosome (43). The IFN-beta enhancer contains overlapping active TFBSs, and the interactions among these TFBSs are insufficient to be characterized by linear and exponential relationships.

We also found that the data sets in which the prediction precision estimated by this study was lower than 0.7 were those of Ulirsch *et al*. assayed in GATA1 OE K562 cells, Ulirsch *et al*. assayed in GATA1 OE K562 cells, Nguyen *et al*. and Shen *et al.*, and all the sequences that were selected were chromosomal segments rather than designed TFBS permutations. This finding also suggests that the transcriptional complexity of chromosomal segments is greater than the complexity of artificial sequences intended for specified purposes.

## Supplementary Material

Supplementary Data
