# Supplementary material for: A new computational method to predict transcriptional activity of a DNA sequence from diverse datasets of massively parallel reporter assays
Source: Nucleic Acids Res. 2017 May 22;45(13):e124. doi: 10.1093/nar/gkx396 (PMC5737609; doi:10.1093/nar/gkx396)
Supplement: Supplementary Data [file gkx396_supp.pdf]

Supplemental Materials

Data pre-processing

Melnikov *et al.*: The transcriptional activity was calculated by the log2 ratios of mRNA tag counts to DNA tag counts, and we removed the samples with 0 DNA tag counts.

Shen *et al.*: In the study of (1), the transcriptional activity was calculated by log2 ratios of mRNA tag counts to DNA tag counts, and in the samples with 0 mRNA tag counts, the pseudo value of 0.001 was set before taking the logarithm. In this study, three experimental replicates of MPRAs were used, and we filtered the samples by setting the cut offs of the standard deviation of the activity at 3.

Sharon *et al.*: The transcriptional activity of (2) was measured by YFP or mCherry expression (see also (2)).

Smith *et al.*: In the study of (3), the three MPRA replicates were contained in the data sets, and we removed the samples with 0 activity and took the average values as the corresponding activity.

Ulirsch *et al.*: In the data set, three replicates of the raw counts of RNA and DNA were provided, and we calculated the transcriptional activity by taking ratios of RNA counts to DNA counts and then by taking the median values of the log2 ratios of three replicates (described by (4) in "http://www.bloodgenes.org/RBC_MPRA/RBC_MPRA_code.html").

Nguyen *et al.*: There are also two replicates of MPRAs and STARR-seq, and we took the average of log2 experimental values as transcriptional activity.

Data process of MPRAs for 18 motifs (Figure 2c and Supplemental figure S5)

First, the data set contains three repeated MPRAs, and we used the log2 of the median values of the experimental replicates as transcriptional activity. Next, we encoded the sequences into TFBS enrichment scores by TRANSFAC, with cut offs that were set to minimize false positive rates, and then we input the explanatory variables into the predictive functions, which were estimated for data set **Nguyen *et al.*** to calculate predictive values. There are 3 types of spacers separating the target motif repeats, and to reduce the influence of spacers, we calculated the average values of the transcriptional activity of three sequences that have different spacers of individual motifs as the activity.

Data process of calculating CREB and RFX activity

In the study of (5), the activities of designed perfect CREB motifs, perfect RFX motifs, two types of 2-bp mutant CREB motifs, two types of 2-bp mutant RFX motifs, full mutant CREB motifs and full mutant RFX motifs were assayed by MPRAs and STARR-seq in normal mouse cortical neurons and in KCL- (potassium chloride-) simulated mouse cortical neurons. We calculated the average activity and standard errors across different conditions for each type of motif (Figure 3b).

TRANSFAC searching

Matrices of vertebrate non-redundant (VNR), liver-specific and fungi with cut offs to minimize the false negative rates of TRANSFAC.201406 were used in this study for these different data sets (see also MATERIAL AND METHODS).

QSAMs

We constructed QASMs by encoding each nucleotide into binary 0-1 variables, for which each position has 3 variables to indicate different nucleotides. The 0-1 variable matrices and corresponding transcriptional activity were performed by multiple linear regressions. Regarding the model of QASM combined with Lasso, we also constructed 0-1 variable matrices as described above, and then, the variable matrices and transcriptional activities were put into Lasso to select variables. Next, the selected sub matrices and transcriptional activities were analyzed with multiple linear regression analysis.

Program packages and parameters

All the R programs were executed using R version 3.3.1 (Supplemental table 4) in the supercomputer system Shirokane3 of the Human Genome Center (HGC), the Institute of Medical Science, the University of Tokyo.

REFERENCES

1. Shen,S.Q., Myers,C.A., Hughes,A.E.O., Byrne,L.C., Flannery,J.G. and Corbo,J.C. (2016) Massively parallel cis -regulatory analysis in the mammalian central nervous system. 10.1101/gr.193789.115.
2. Sharon,E., Kalma,Y., Sharp,A., Raveh-Sadka,T., Levo,M., Zeevi,D., Keren,L., Yakhini,Z., Weinberger,A. and Segal,E. (2012) Inferring gene regulatory logic from high-throughput measurements of thousands of systematically designed promoters. *Nat. Biotechnol.*, **30**, 521–530.
3. Smith,R.P., Taher,L., Patwardhan,R.P., Kim,M.J., Inoue,F., Shendure,J., Ovcharenko,I. and Ahituv,N. (2013) Massively parallel decoding of mammalian regulatory sequences supports a flexible organizational model. *Nat. Genet.*, **45**, 1021–8.
4. Ulirsch,J.C., Nandakumar,S.K., Wang,L., Giani,F.C., Zhang,X., Rogov,P., Melnikov,A., McDonel,P., Do,R., Mikkelsen,T.S., *et al.* (2016) Systematic functional dissection of common genetic variation affecting red blood cell traits. *Cell*, **165**, 1530–1545.
5. Nguyen,T.A., Jones,R.D., Snavely,A.R., Pfenning,A.R., Kirchner,R., Hemberg,M. and Gray,J.M. (2016) High-throughput functional comparison of promoter and enhancer activities. *Genome Res.*, **26**, 1023–1033.

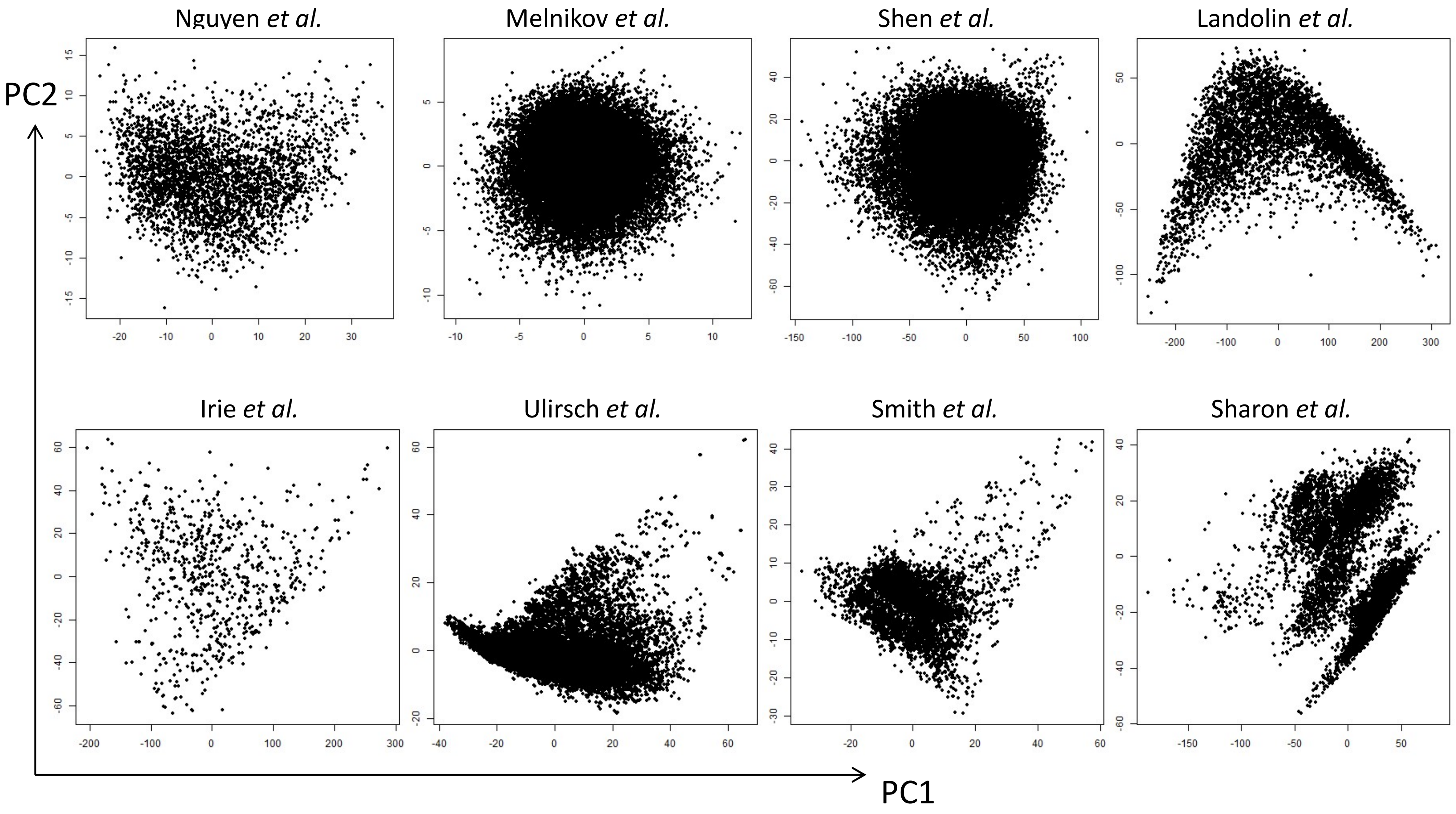

Supplemental figure S1: PCA projection of TFBS enrichment scores onto PC1 and PC2 for different data sets.

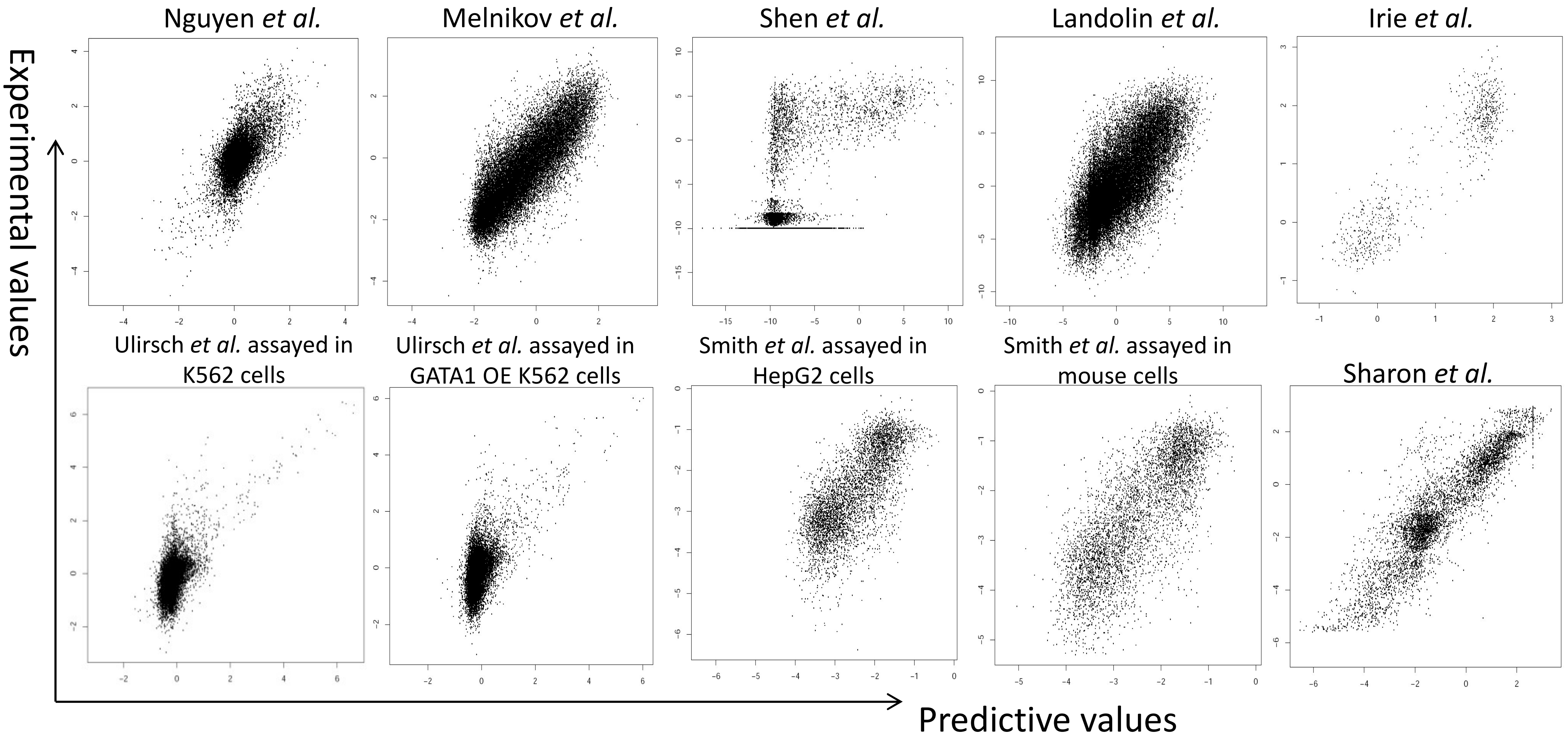

Supplemental figure S2. Scatter plot of closed test for different data sets.

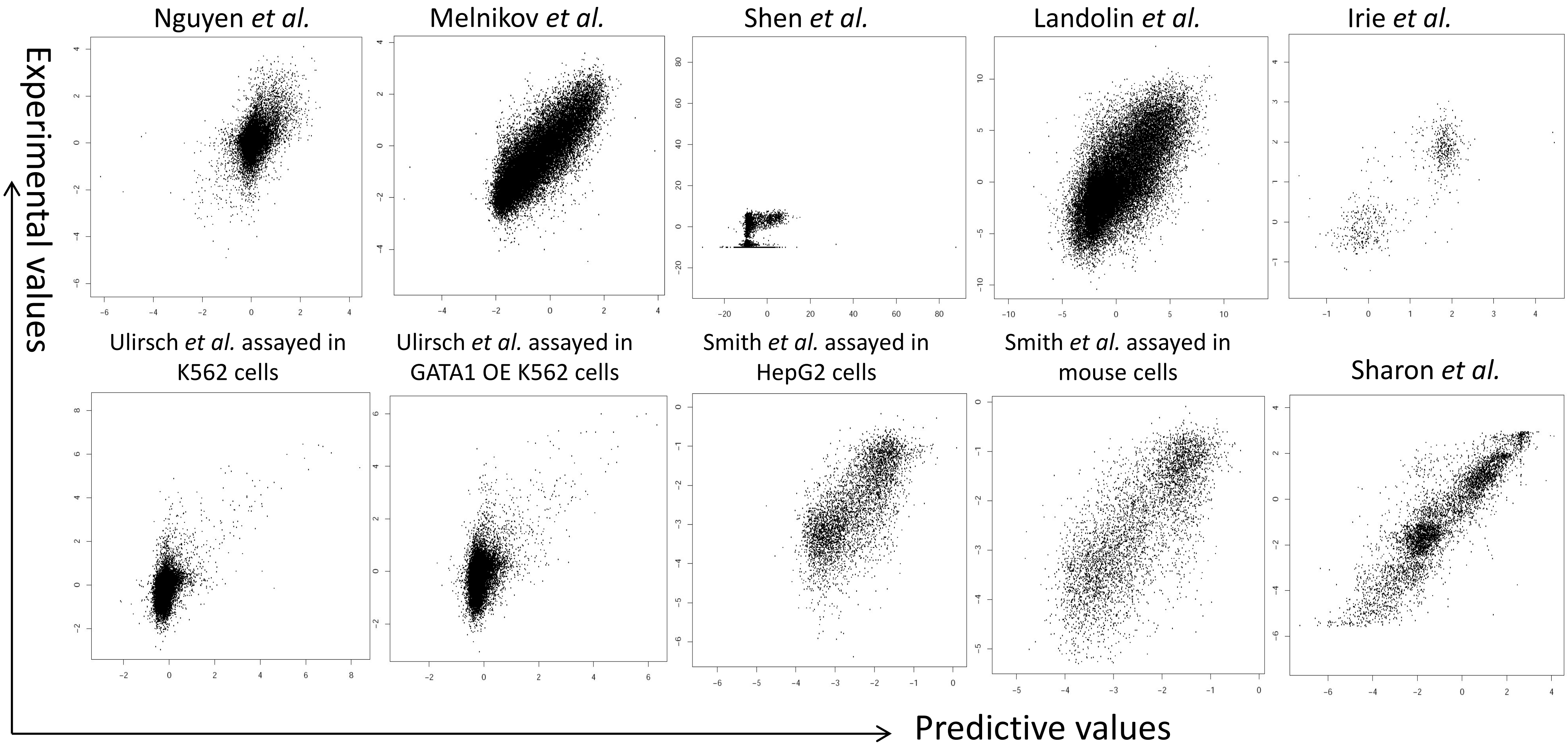

Supplemental figure S3. Scatter plot of open test for different data sets.

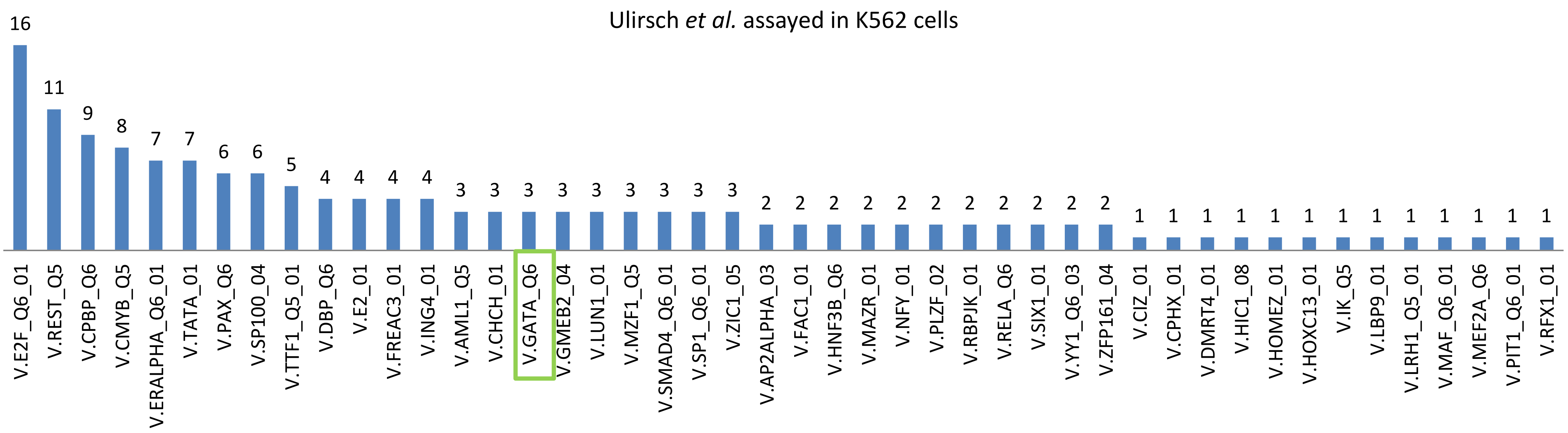

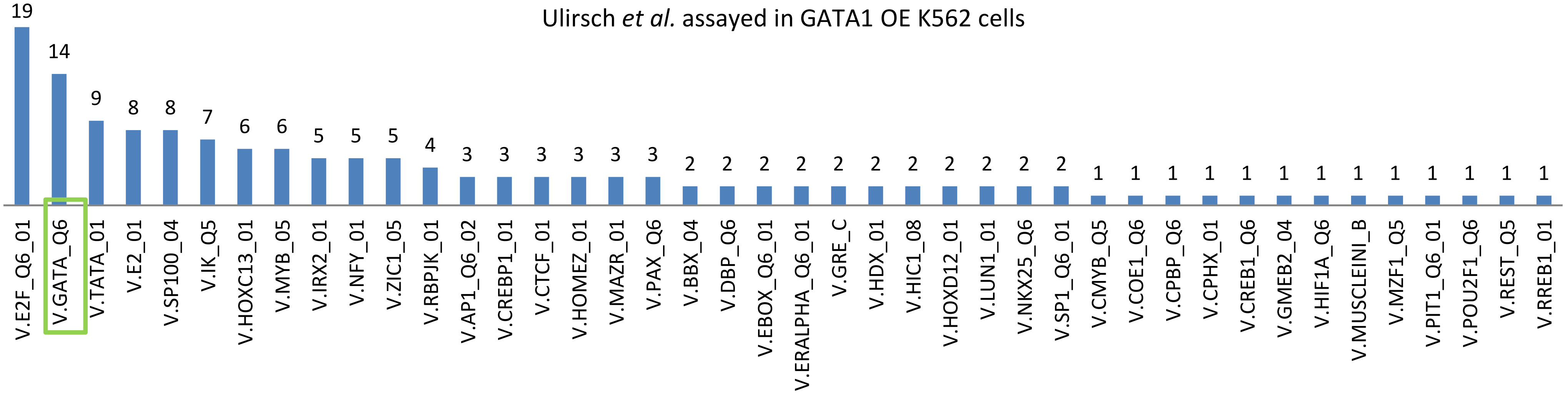

Supplemental figure S4. TFBS frequencies across all predictors for the predictive functions from Ulirsch *et al.* assayed in K562 and GATA1 OE K562 cells.

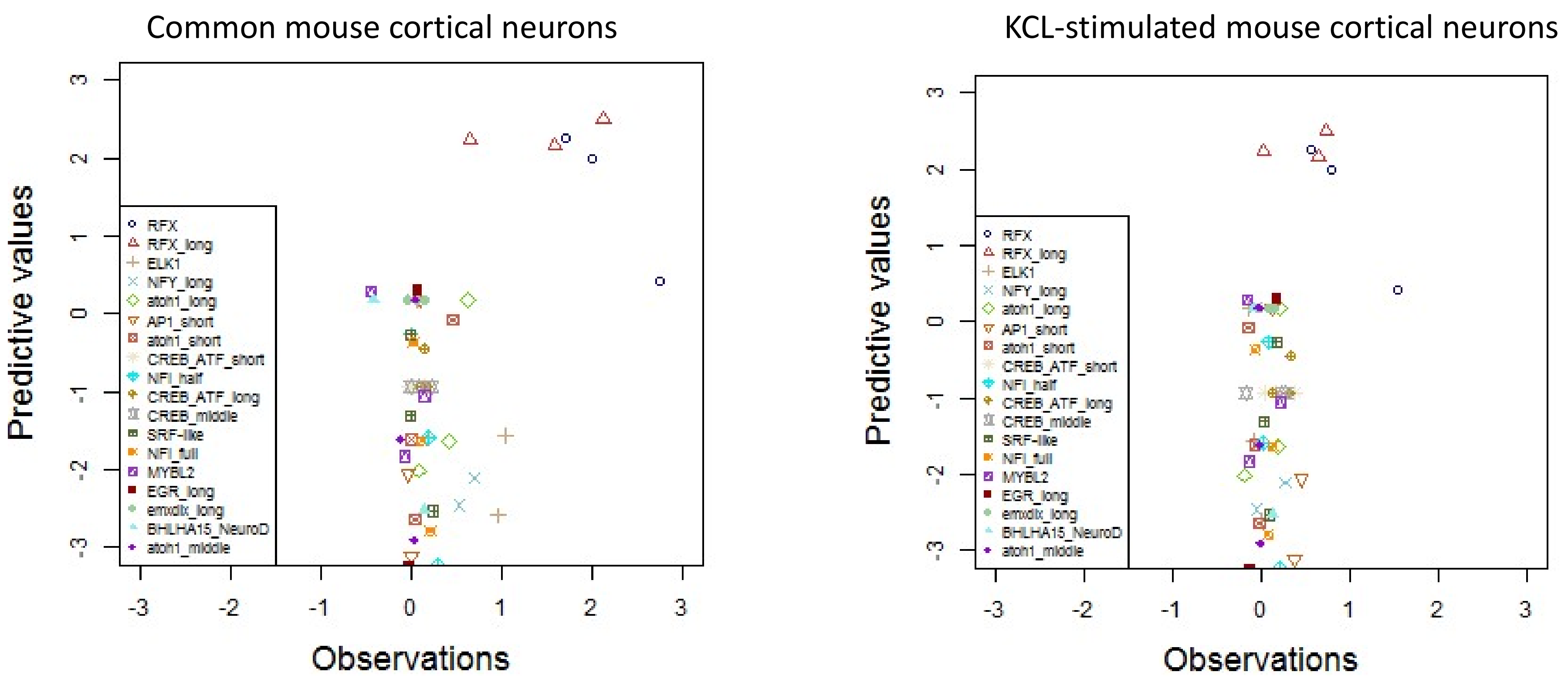

Supplemental figure S5. For 54 individual sequences of 18 selected motifs (see also Figure 3d), we determined correlation coefficients between the predicted values and experimental values of 0.40 and 0.47 for control and KCL-simulated mouse cortical neurons, respectively. Because the artificial sequences were specifically designed, a number of these sequences (6/18 motifs) with a length of 6-8 bp had high sequence similarities due to corresponding motifs that were much shorter than spacers, and we took the average to reduce the influence of the spacers.

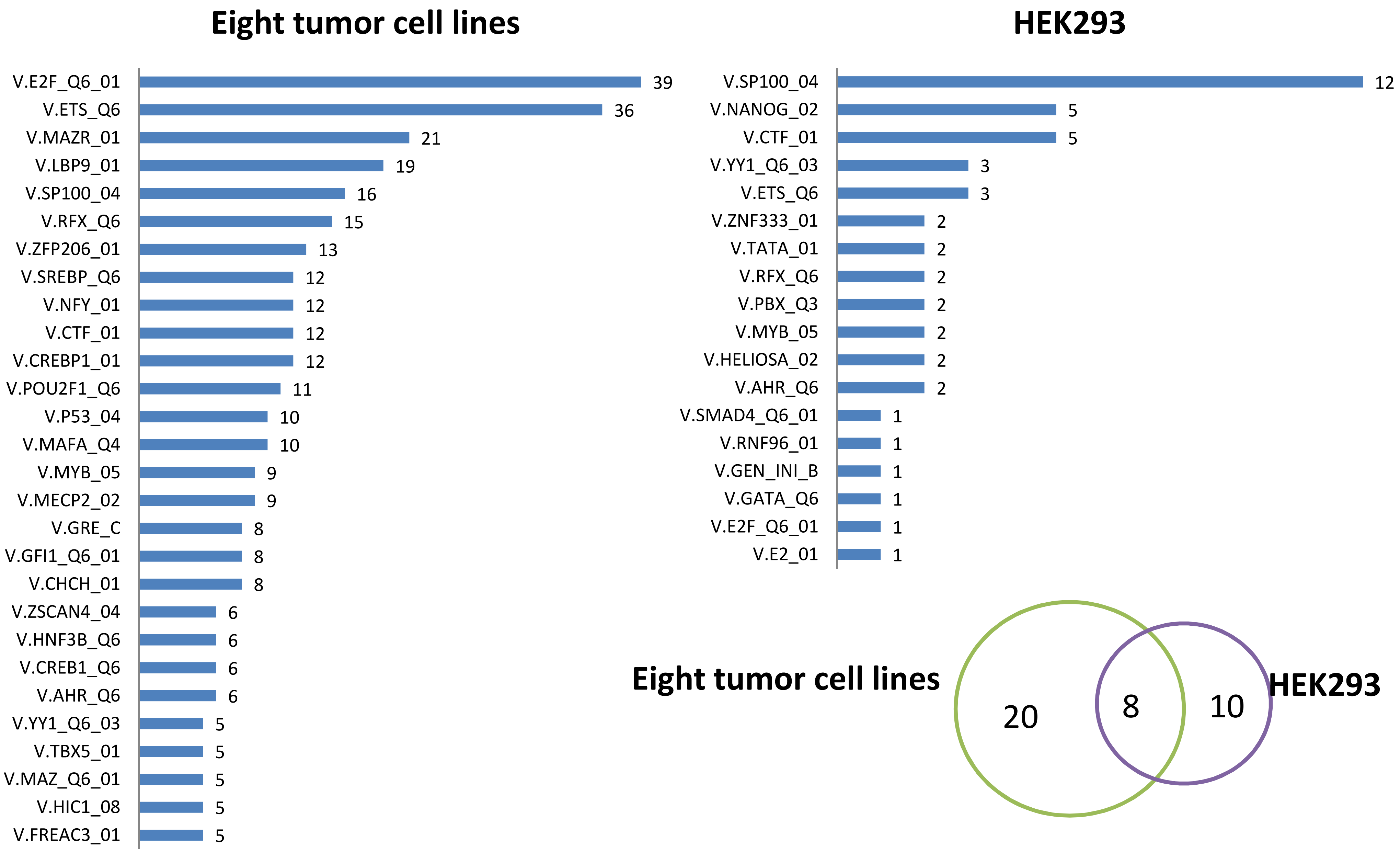

Supplemental figure S6. Selected TFBSs and the corresponding frequency. (Left) Frequency of selected TFBSs that occur >=5 times in the predictors within the predictive functions estimated by the modeling data set of Landolin *et al.* (Right) Frequency of all the selected TFBSs of Irie *et al.* and the number of selected TFBSs of Landolin *et al.* (frequency>=5) and Irie *et al.*

Supplemental Figure S7: Analysis of Figure 2c removing two RFX motifs.
(a,b) Scatter plots of predictive values and observations of 16 samples which removed two RFX motifs in Figure 2c. (c) Three types of correlations of predictive values and observations of 16 samples showed in (a). (d) The distribution of experimental activities of the 16 data points. The correlation coefficients are dragged by the high transcriptional activities of two RFX motifs ("RFX" and "RFX_long" in Figure 2c) and the correlation coefficients were dramatically decreased, if the two RFX motifs were removed (showed in the a and b). We also obtained similar results when we calculated the correlation by other methods (Spearman's rho and Kendall's tau in the table c). It is probably due to the fact that the remaining data points represent very low expression levels. The distributions of experimental activities of the data points after removing the two RFX motifs are shown in the table d. The expression levels of (the 3rd: the max quantiles) for the datasets of "mouse cortical neurons" and "KCL stimulated mouse cortical neurons" are (0.16: 0.69) and (0.11: 0.32), respectively. Those values are very small compared to the other datasets. As far as those lowly expressed points are concerned, there might be different feature patterns between the trained datasets (genomic segments) and the predicted datasets (designed sequences). Thus, only limited accuracy was obtained for the proposed method. Indeed, when we limited the data points to those of relatively high expression levels, the correlation was more significant. Namely, the Pearson's r of the 5 sequences showing the highest transcriptional activities (indicated by the circle in the following figure a) was approximately 0.56. On the other hand, no significant correlation was observed for the figure b, probably because all the data points were less than 0.5 (in the observed expression level represented on the X-axis).
We could not precisely explain the cause of the lack of accuracy. It may be derived from the fact that the present method only considered the TFBS enrichments and ignored other information such as their positions or orientations, which might play a more important role in determining the very low expression levels. In any case, it is questionable to infer the biological relevance of sequences of such low activities. With higher sensitivity of MPRA assays, they may have detected transcriptional activities for those that have no or noise-level transcriptions.

a

Mouse cortical neurons

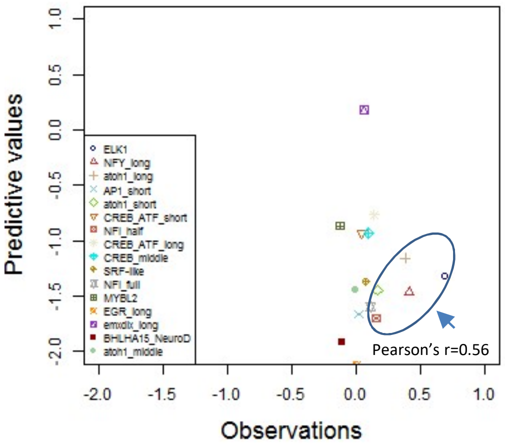

b

KCL-stimulated mouse cortical neurons

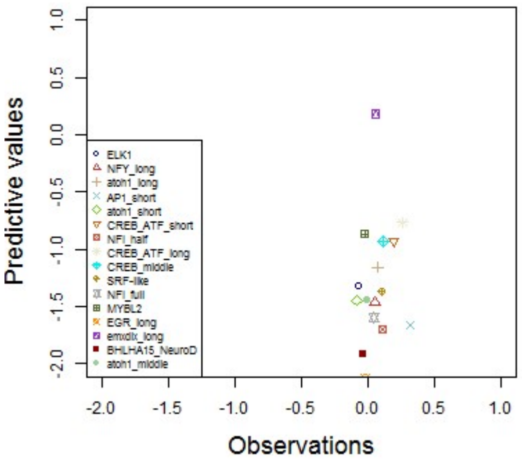

c

	Pearson's r	*Spearman's rho*	*Kendall's tau*
normal	*0.02*	*0.08*	*0.06*
KCL	*0.21*	*0.25*	*0.19*

d

	min	*1st quantile*	*mean*	*3rd quantile*	*max*
normal	*-0.12*	*0.02*	*0.13*	*0.16*	*0.69*
KCL	*-0.08*	*-0.02*	*0.07*	*0.11*	*0.32*

Data set	This study			MLR			Lasso			BQR			QSAM			QSAM+Lasso		
	Closed test	Open test	# of predictors	Closed test	Open test	# of predictors	Closed test	Open test	# of predictors	Closed test	Open test	# of predictors	Closed test	Open test	# of predictors	Closed test	Open test	# of predictors
Ulirsch *et al.* assayed in GATA1 OE K562 cells	0.55	0.49	16 - 30	0.44	0.42	160	0.42	0.41	64	0.32	0.30	161	0.46	0.41	435	0.44	0.41	208
Ulirsch *et al.* assayed in K562 cells	0.57	0.50	21 - 26	0.44	0.42	160	0.42	0.41	53	0.32	0.29	161	0.50	0.45	435	0.47	0.45	212
Nguyen *et al.*	0.64	0.50	21 - 36	0.40	0.38	164	0.37	0.36	69	0.26	0.23	165	0.31	0.22	414	0.25	0.19	139
Shen *et al.*	0.64	0.52	20 - 48	0.40	0.39	161	0.40	0.38	134	0.35	0.34	162	-	-	-	-	-	-
Smith *et al.* assayed in HepG2 cells	0.73	0.71	28 - 28	0.67	0.66	94	0.66	0.65	40	0.60	0.58	95	0.67	0.56	504	0.62	0.55	261
Landolin *et al.*	0.73	0.70	21 - 50	0.64	0.64	171	0.64	0.64	122	0.60	0.60	172	-	-	-	-	-	-
Smith *et al.* assayed in mouse cells	0.78	0.76	35 - 35	0.71	0.69	94	0.70	0.69	43	0.64	0.63	95	0.73	0.64	504	0.69	0.63	277
Melnikov *et al.*	0.83	0.81	25 - 47	0.79	0.79	158	0.78	0.78	78	0.75	0.75	159	0.79	0.79	261	0.79	0.78	196
Iriel *et al.*	0.92	0.85	28 - 28	0.87	0.79	160	0.85	0.81	86	0.65	0.55	161	-	-	-	-	-	-
Sharon *et al.*	0.92	0.91	16 - 30	0.90	0.89	353	0.88	0.87	145	0.86	0.84	354	0.86	0.83	309	0.84	0.83	209

Supplemental table 1. The correlation coefficients between the predictive values and experimental values of the closed test and open test as well as the corresponding number of predictors for different methods.

Predictor-associated enhancer specific activity	Coefficients	Cluster label
kclEnh*h(0.958-V.CEBPA_Q6)	-1.08	14
kclEnh*h(1.883-V.ZFX_01)	0.10	13
kclEnh*h(11.239-V.ZIC1_05)	0.12	4
**kclEnh*h(V.AP1_Q6_02-3.545)**	**0.59**	**14**
kclEnh*h(V.BEN_01-15.139)	-1.55	9
kclEnh*h(V.CPBP_Q6-4.96)	-0.09	4
kclEnh*h(V.SP100_04-3.694)	-0.15	4
kclEnh*h(V.SP100_04-6.888)	-0.12	7
kclEnh*h(V.ZFP161_04-4.641)	-0.12	4
kclEnh*h(V.ZFX_01-1.883)	-1.31	13
kclEnh*h(V.ZIC1_05-11.239)	-0.19	4

Supplemental table 2. Predictors that show enhancer activity preferences and the estimated coefficients and corresponding cluster (cluster labels are shown in Figure 3b).

Predictor	Coefficient	Cluster label
h(4.777-V.GATA_Q6)*h(24.793-V.TATA_01)	-0.00155	3
h(V.COE1_Q6-0.864)*h(V.GATA_Q6-2.681)	2.73067	4
h(V.GATA_Q6-2.681)*h(0.733-V.RREB1_01)	2.008507	4
h(V.GATA_Q6-1.807)*h(V.REST_Q5-0.807)	5.185114	6
h(1.807-V.GATA_Q6)*h(V.HOXC13_01-9.591)	-0.03376	6
h(1.807-V.GATA_Q6)*h(9.591-V.HOXC13_01)	-0.06378	6
h(V.AP1_Q6_02-3.87)*h(1.807-V.GATA_Q6)	-0.73253	6
h(V.GATA_Q6-1.807)*h(V.RBPJK_01-3.398)	0.777971	6
h(V.GATA_Q6-1.807)*h(3.398-V.RBPJK_01)	-0.1473	6
h(V.CREBP1_01-4.421)*h(V.GATA_Q6-1.807)	0.491494	6
h(4.421-V.CREBP1_01)*h(V.GATA_Q6-1.807)	0.244953	6

Supplemental table 3. Predictors of Ulirsch *et al.* assayed in GATA1 OE K562 cells that take the forms of the hinge function of other TFBSs multiplied by the hinge function of the GATA family binding site (V.GATA_Q6). Coefficients were estimated by MPRS, and the cluster labels are shown in Figure 3c.

Algorithm	R package	Method	Specified parameters
Regression tree	rpart	rpart	Control.rpart (minbucket=*minbucket*) (see Methods as well);<br>cp=0.01(for multiple conditions data, cp=0.005)
MARS	earth	earth	degree=2
MLR	base	lm	default
Lasso	glmnet	glmnet	s=0.01
BQR	bayesQR	bayesQR	quantile=0.5

Supplemental table 4. R packages used in this study and the corresponding parameters

	Mouse	HepG2	Binding TFs
V.AHRHIF_Q6	1		AhR
V.ATF4_Q6	3	5	ATF-4
V.CREBATF_Q6	3		CREB, ATF
V.FOS_01	2		c-Fos
V.FOS_02	6	1	c-Fos
V.FOS_05	1		c-Fos
V.FOXA1_02		1	FOXA1
V.FOXA1_03	1		FOXA1
V.FOXA1_06	2	4	FOXA1
V.FOXA2_04	3		FOXA2
V.FOXA2_05		7	FOXA2
V.FOXA2_06	1		FOXA2
V.HIF1A_Q6		1	HIF-1A
V.HIF1AARNT_01		2	HIF1A,ARNT
V.HNF1_C		2	HNF-1A
V.HNF1_01	2		HNF-1A
V.HNF1_Q6_01	2		HNF-1A
V.HNF1A_01	7	5	HNF-1A
V.HNF1B_01		3	HNF-1B
V.HNF1B_Q6	1		HNF-1B
V.HNF3A_Q6	4		HNF-3A
V.HNF3G_Q4		3	HNF-3G
V.HNF4A_02	1		HNF-4A
V.HNF4A_04		1	HNF-4A
V.HNF4A_09	1		HNF-4A
V.HNF4A_10	2		HNF-4A
V.HNF4ALPHA_Q6	7		HNF-4A
V.HNF4DR1_Q3	2		HNF4 family
V.HNF6_Q4		2	HNF-6
V.LFA1_Q6		2	HNF-1B
V.NFKAPPAB50_01	2	2	NF-kappaB
V.NR2F1_04	2		NR2F1
V.USF1_Q4		1	USF1

Supplemental table 5: Frequencies of TFBSs selected by the response functions of the data sets of Smith *et al.* assayed in HepG2 and mouse cells
